# What prompts young adults in Ireland to attend health services for STI testing?

**DOI:** 10.1186/1471-2458-9-311

**Published:** 2009-08-26

**Authors:** Myles Balfe, Ruairi Brugha

**Affiliations:** 1 Department of Epidemiology and Public Health Medicine, Royal College of Surgeons in Ireland, Dublin, Republic of Ireland

## Abstract

**Background:**

In-depth understanding of the factors that prompt young adults to attend health services for sexually transmitted infection (STI) testing are needed to underpin sexual health programes. We conducted a qualitative study to identify and explore why young adults (18–29 years) in Ireland attended specialist and community health services for STI testing; the factors that supported/undermined their decisions to seek STI testing; and any factors that led to delay in seeking STI testing.

**Methods:**

Semi-structured interviews with 30 adults (21 women, 9 men). Young adults were recruited from General Practice (GP) practices, Third Level College health services, Family Planning clinics and specialist STI treatment services for men who have sex with men (MSM). Interview questions examined why respondents decided to go for STI testing, whether they acted upon this desire immediately or decided to wait, and what they felt were important barriers/enablers to their health-seeking attempts. Interviews were thematically analyzed using standard qualitative techniques.

**Results:**

Respondents sought STI testing for one of four reasons: they had reached a transitional moment in their lives (they were either about to stop using condoms with their sexual partner or were emerging from a period of their lives where they had a series of risky sexual relationships); they had had unprotected sex with a casual partner; they had symptoms of infection; and/or they were required to do so by their employer. Catalytic factors included media and government health promotion campaigns and knowing someone with an STI. However, many respondents delayed seeking testing. Reasons included respondents' concerns about stigma and that they would be judged by healthcare professionals, and feelings of invulnerability. Importantly, several respondents who waited up to four weeks to make an appointment after their initial decision to seek STI testing did not view this as delay.

**Conclusion:**

Sexual health promotion campaigns for young people should address the reasons why they delay testing, specifically through measures to avoid stigma (supply-side) and reassure young adults (demand-side). Strategies to increase testing-uptake should focus on these four key opportunities – young adults leaving relationships, those entering relationships where condoms will not be used, those who have had unprotected sex and those with STI-related symptoms.

## Background

Sexually transmitted infections (STIs) are increasingly important public health issues in the Republic of Ireland, because of the associated risks of infertility, pelvic inflammatory disease (PID) and cancer [1-3]. The number of diagnosed STI cases reported to the Irish Health Protection Surveillance Centre (HPSC) increased from 2228 in 1989 to over 10,500 in 2003, with the rate of increase being particularly high amongst young adults [4]. The increase in incidence, in a country that does not have a systematic population screening programme, reflects better detection techniques, increased opportunistic screening by healthcare professionals; and also proactive decisions by young people to seek STI testing in an environment that is more open to acknowledging these risks. Given that such willingness provides an opening for promoting personal and public health, remarkably little is know about the factors and influences that prompt young people to seek STI testing. Most of the international research on young adults' STI testing practices has focused on barriers rather than on enablers and reasons that encourage them to seek testing [5,6].

The small number of studies that have examined young adults' STI care-seeking have found that it is influenced by factors including: health service characteristics (clinic opening hours, cost), patient characteristics, recognition by individual that they have engaged in 'risky' sexual practices; and sometimes real or suspected symptoms [7]. Researchers [7-10] have argued, consequently, that more work is needed to understand the health seeking activities of young adults in relation to STIs, especially in countries and amongst populations where STI research and systematic screening have been historically lacking [11]. Understanding young adults' STI-related care-seeking behaviours is also central to population approaches to controlling the spread of STIs [11]. Better evidence could assist policy makers, programme managers and clinicians in funding, designing and implementing health promotion campaigns that address young people's concerns about STI testing processes [7] and thereby encourage demand or client-led STI screening practices [7,8,12]. Systematic population screening is often preferable, especially for largely asymptomatic STIs such as Chlamydia. However, where they are not feasible (Ireland lacks unique health service identifiers at the population level), it is essential to maximize prompt testing-seeking in those at greatest risk of having and transmitting a STI.

Drawing on qualitative interviews with thirty young adults (18–29 years) who had previously attended either specialist STI services or community health-care settings for STI testing, in this paper we explore the following questions:

• What prompts young adults in Ireland to attend health services for STI testing?

• Do young adults delay in following through on their decision to seek STI testing, and if so, why?

• What factors support/undermine young adults' decisions to seek STI testing?

## Methods

Thirty in-depth interviews were conducted with young adults who had previously attended health services for STI testing in the Republic of Ireland. Respondents were recruited from General Practice (GP) practices, Third Level College health services, Family Planning clinics and specialist STI treatment services for men who have sex with men (MSM). We utilized a qualitative approach because we wanted to explore young people's perspectives in detail. Moreover, the dearth of research on these issues and the hypothesized complexity of young adults' STI care-seeking practices demanded an exploratory method. Qualitative methods have proven efficacy in sexual health research [5].

Respondents were eligible to take part in the study if they were between eighteen and twenty nine years of age (the age group with the greatest increase in detected incidence of STIs in the Republic of Ireland over the previous decade) [4]. We purposely sampled respondents in order to recruit both men and women, respondents of different ages (late teens, early 20 s, mid 20 s, late 20 s), and respondents who had sought STI testing from specialist and community-healthcare settings. Twenty one respondents were female, nine were male. Ethical approval for the study was received from the Royal College of Surgeons in Ireland's ethics committee, and from the ethics committee of the Irish College of General Practitioners.

Respondents were recruited either by the first author or by a healthcare professional working in the healthcare setting. Potential respondents were provided with an information leaflet, which provided background information on the study, what participation would involve (a one hour interview focusing on respondents' reasons for attending services for STI testing); their freedom to avoid issues and stop the interview at any point; and assurances on anonymity and how information would be recorded and used. Participating respondents were asked to respond 'yes' by text and were given the option of completing the interview by telephone or face-to-face. Twenty four respondents chose to do the interview by phone and six face-to-face. Respondents who completed face-to-face interviews were asked to give written consent to take part in the study; respondents who completed telephone interviews were asked to give verbal consent.

A semi-structured interview approach was used. Interview questions examined why respondents decided to go for STI testing, whether they acted upon this desire immediately or decided to wait, and what they felt were important barriers/enablers to their health-seeking attempts. Follow-up questions explored the reasoning behind respondents' responses in more detail. A non-directive approach was used in the interviews to allow respondents to shape their own accounts. Interviewing continued until we deemed theoretical saturation to have been reached (the point at which no new themes were emerging). Researchers have proposed 30 as an approximate or working number of interviews at which one could expect to be reaching theoretical saturation when using a semi-structured interview approach [13].

Interviews were tape-recorded (with respondents' permission) and fully transcribed. Significant key words, phrases and themes in the interview transcripts were marked with summary words or 'codes' by the first author. Coded transcripts were then passed on to the second author. The second author reviewed the coded transcripts to ensure the reliability of the first author's codings, and to see if any further themes could be detected in the transcripts that the first author had missed. Once the first and second author agreed on the codings, all codes that were thematically similar were grouped together, and labeled with a summary code, called a category. In line with standard qualitative procedures [7], these categories then became the organizing themes of our analysis. In the findings section representative quotations are accompanied by codes providing details about the respondents who uttered those quotations (see Table 1 for a key to these codes).

**Table 1:** Key for respondent quotation codes

M	Male (self-defined as heterosexual n = 6, MSM n = 3)
F	Female
Early/mid/late 20 s	Respondents' age
SH	Went for STI testing in Student health service
FP	Went for STI testing in Family Planning service
GP	Went for STI testing in GP practice
STI	Went for STI testing in STI clinic
MSM	Went for STI testing in specialist clinic for men who have sex with men (MSM)
-	Tested negative for STI
+	Tested positive for STI

## Results

### Why do young people decide to go for STI testing?

Respondents attended health services for STI testing for one or more of four reasons. First, they had come to a *transitional moment *in their lives or personal relationships. This could be where respondents were about to enter a more intimate, though potentially more hazardous, phase in a sexual relationship (typically when they decided to stop using condoms for the first time) and wanted to minimize physical vulnerability and feelings of uncertainty.

I was maybe 24 and I was just moving in with a guy and we'd bought a house and we were going to be having unprotected sex and going on the pill so I said, right, I'm getting everything checked out. (F/FP/late 20 s/+).

I'm with a long term girlfriend at the moment and we don't want to use condoms any more and she got tested and I got tested. We wanted to be sure that we were both clean and that we wouldn't be giving anything to each other. (M/SH/early20 s/-).

A transitional moment could also be an instance where respondents were emerging from a sexually risky phase of their lives. This could be a phase where respondents had had sex with a number of potentially risky partners, or when they had been in a long-term sexual relationship with a partner whom they retrospectively considered to be 'high risk'.

I was traveling abroad for a few months. I split up from my girlfriend and let myself loose being free and single again. I had a number of partners in a fairly short period of time. I felt safe. They weren't prostitutes and they weren't working in strip clubs. There's a lot of diseases with hookers and stuff like that but they were just ordinary girls that I knew from college. It was in the back of my mind that maybe there was a chance [that could get STI] so I just decided to get the test. (M/SH/mid 20 s/-).

STI testing at the exit phase of a risky relationship or life period had ritualistic and moral significance for respondents. It served to bracket the risky period/relationship and enable and mark the beginning of a new and clean identity [14], one characterized by self-respect and a protective, positive orientation towards self and body. The process of going for a STI test was portrayed as a 'cleansing' in itself.

For about a year before I had been going through a really bad time, I went out with a few different guys and stuff and just when I came through that I was kind of looking back on some of the crazy stuff that had happened in the previous year and I just wanted to completely cleanse myself of everything that had happened. I just thought, OK I suppose a part of that is going to have to be making sure that everything is OK. I don't know, I just wanted to cleanse myself of everything that had happened in that year. Just be able to close the chapter. Gone. (F/STI/mid 20 s/-).

I wasn't in a great position in my life and didn't really care too much and there was one or two times where I didn't use protection. There was a month or two where I just kind of realized the direction I was going and figured that if I wanted to make anything of my life I needed to cop myself on and start focusing on what I was doing. One part of it was to get checked out and start moving on from there. So that was what got me in the mentality of getting it done. I think that was the fundamental part of it to me. That it would help me start again. (M/SH/mid 20 s/-).

The second reason that respondents went for STI testing was that they had *unprotected sex *with an unknown partner.

I just had a one night stand with somebody and I didn't use protection. And I just wanted to make sure. Cos like you never know. I don't know anything about that person or, you know. (F/GP/mid 20 s/-).

I'd had unprotected sex and just thought I should get checked. I didn't have any symptoms but I still kind of worried about it. (M/SH/early 20 s/-).

Respondents (especially female respondents) typically experienced unprotected sex with an unknown partner as a catastrophic event, triggering strong feelings of anxiety, shame and guilt. Unprotected sex undermined respondents' identities, their sense of who they were as individuals. One respondent for example noted:

I just did this [had unprotected sex], my God, what kind of person am I? (F/GP/early 20 s/-).

Unprotected sex catapulted several respondents into a state of vulnerability, triggered by uncertainty over the body's risk status.

I was quite nervous cos like you don't know. I wasn't sure like, had I got anything, had I not got anything. Any other times I've gone since I've known I've been safe but that time because I knew I'd had unprotected sex I was quite unnerved by the fact that I could have something. (Female/GP/early 20 s/-).

STI testing provided emotional reassurance, and a means of stitching a 'respectable' identity back together by enabling respondents to feel that they were risk-avoiding responsible individuals who looked after their bodies.

Interviewer: And how did you feel about yourself after you had the test?

R: Better. Like I was someone who had done something right (F/GP/early 20 s/-).

The third reason for going for testing was the presence of *unusual symptoms *in or near the genital region. Respondents were often unsure of what these symptoms were, and sometimes did not expect them to be symptoms of an STI. As such these respondents did not necessarily attend health services for STI testing. Most of the symptoms that respondents reported were described as being 'marks' or 'spots'. Symptoms that would indicate discharge from the vagina or penis were rarely mentioned.

I kind of had a mark down there and I was unsure of it you know. (M/GP/late 20 s/-).

My background would have been that it was symptoms in the genital area so that's why I decided. I hadn't a clue. (F/STI/late 20 s/+)

Obviously they came up like spots down there. I got sore and red. I actually didn't think it was going to be an STI. I'd never really heard about them. I thought it was just a rash so I said it to my sister and my mum not really thinking much about it. (F/GP/early 20 s/+).

I had symptoms that I would attribute to an STI like increased frequency of weeing or stuff like that. (F/SH/late teens/+).

The final reason for attending a health service for STI testing was *being required to by an employer or by a state*. Of the four reasons provided here for attending a health service for STI testing this was the most meaningless for respondents, as it was the most disconnected from their personal practices, biographies and emotions.

I just went into the doctor and I said I need a visa for working abroad, can you give me a HIV test. I didn't see that test as having any personal significance. It was just a piece of paper that I needed. (F/STI/mid 20 s/-).

Most respondents who sought testing wanted to receive a 'full screen', that is to be tested for all of the most common sexually transmitted infections. As noted in the next section, several respondents were particularly concerned about being tested for specific sexually transmitted infections such as Chlamydia (the young gay men who took part in the study (n = 3) were the only respondents to be concerned about Syphilis, and were the most concerned about contracting HIV); however these respondents, too, wanted 'full screens'. Respondents themselves (prior to completing testing) were often unsure about all of the STIs that they could be tested for. Analysis of interview transcripts reveals that most respondents were tested for HIV/AIDS, Gonorrhea, Genital Warts, Herpes, Non-Specific Urethritis (NSU), Chlamydia, Syphilis, Hepatitis and Trichomonas. Respondents who tested positive for STIs were all diagnosed with Herpes, Chlamydia or NSU.

### Factors encouraging testing

Several factors encouraged respondents' decision to seek STI testing. One was seeing STI testing as a responsible practice that adults should engage in.

I'm nearly 28. I think it's just kind of the mature thing to do. (F/STI/late 20 s/-).

I'm a grown up. It wasn't something I would have even thought of when I was younger. (F/FP/late 20 s/+).

Another was having a protective orientation towards partners and other young adults. All respondents' indicated that they would experience significant guilt and distress if they transmitted an STI to another individual. STI transmission was viewed as a fundamentally unjust activity, a violation of moral norms.

I'd feel like shit, absolutely [if I passed on a STI]. And that's why I wanted to get screened as well. I didn't want to do that cos I've had sexual partners in the past and she hadn't. I suppose it's like a domino effect, it's like passing it onto somebody and they in turn pass it on to somebody else. It's kind of unfair. (M/SH/early 20 s/-).

Respondents (male and female) expressed particular concerns about the damage that STIs could do to their future fertility, indicating awareness of important complications in this at-risk group. STI testing was seen a crucial means of protecting this fertility.

I just wanted to make sure I didn't have anything. I got a full testing like. Just for peace of mind. I could end up not being able to have children or like, I don't know, getting someone else not able to have to children (F/FP/late teens/-).

The perceived risk status of respondents' sexual partners was also a factor that influenced care-seeking. Respondents judged partner's risk based on a number of characteristics, including appearance and geographical origin. As such they sometimes tended to rely on visual cues when judging partners' risk status. 'Dirty' partners or those from perceived risky regions (especially Africa and South America) were most likely to increase respondents' concerns about the need to seek STI testing. Partner's past sexual history also influenced their care-seeking behavior; however in many cases respondents had only a vague notion of their partner's actual sexual history other than that it had been 'colourful'.

If they had AIDS or herpes I presume I would have noticed something. But this one was so random and drunken and stupid. And I don't know the guy, he was from Africa. I know that's an awful preconception but if he didn't want to use a condom with me I presume he didn't use one with anyone else. (F/GP/early 20 s/-).

I possibly didn't know as much about his partners or how many there might have been. (F/GP/mid 20 s/-).

Many respondents who did not have symptoms when they attended health services testing expressed anxieties about the risk that asymptomatic STIs (especially Chlamydia) could affect them without their knowledge.

I've heard that there's no symptoms with Chlamydia and I've no symptoms so I thought that maybe if I do have one, this could be it (M/SH/early 20 s/-).

Chlamydia is such a scary, scary thing now, cos it's you know asymptomatic (F/GP/late 20 s/-).

Respondents' anxieties about symptomless STIs stemmed from two sources. The first of these was consciousness-raising health promotion materials.

I remember at the time they were doing a thing on Hollyoaks [British soap opera for young adults]. They were doing the whole STI thing on the TV. It was basically what I got from there. (F/GP/late 20 s/+).

You are aware, you see the signs around and advertising on TV and you are aware that unprotected sex is not entirely beneficial for you! And the risk of catching an STI is quite a real risk nowadays (F/GP early 20 s/-).

The second was proximity to individuals who had been diagnosed with STIs.

And I know a girl who had it at one stage so. When someone in your circle of friends has had it you're thinking well like maybe it's more common than a lot of people think. That was the only reason I was thinking it. (M/SH/early 20 s/-).

Just that somebody close to me had done it and it was grand. It kind of brought it more down to earth rather than something that gets done. You know what I mean like. It was always there, this STI check but now somebody had actually gone for it in DIT as well, so it was close by. It was more real than something I just saw on a poster. (M/SH/early 20 s/-).

Together health promotion materials and contact with individuals who had attended for STI testing appeared to disrupt what sociologist Anthony Giddens [15] referred to as respondents' 'protective cocoons', the psychological filters that they used to screen out threatening anxieties. These included thoughts that they could have, or might be at risk of having, STIs, and which they used to maintain feelings of invulnerability and 'personal specialness'. Health promotion and by-proxy awareness of STIs interfered with the psychologically protective delusions that respondents might otherwise have had about STIs, such as 'only 'bad' people get STIs' and 'the chance of contracting an STI is miniscule'. They revealed to respondents that many people contracted STIs, including people 'like them'.

### Delayed testing

A number of respondents immediately followed through on their decision to go for testing. The feature that all of these respondents had in common was an immediate, pressing anxiety to know if something was wrong with their bodies. This stemmed from a perceived high risk that they could have an STI.

I kissed a fella who had syphilis. I was like to him, I'm not being bad but I have to get checked now (M/MSM/late 20 s/-).

I had to find out what it [symptom] was. (M/GP/late20/-).

Most respondents, though, delayed to varying extents on following through on their decision to attend a health service for STI testing. This'procrastination period could last from several weeks to, in one instance, over seven years. Anxieties about the potential social consequences of being diagnosed with an STI were common reasons for delay. Respondents felt that STIs were highly stigmatised infections in Ireland and that even being known to attend a health service for testing could discredit young adults' identities.

People are afraid of the consequences of what's going to happen if you do have this thing. (F/FP/late 20 s/+).

Feelings of shame and embarrassment encouraged delay. These feelings could be associated with the STI testing process, such as having to take clothes off in front of healthcare professionals (this concern was much stronger amongst female respondents).

The reason I hadn't gone before was because of plain mortification. Because of stripping off for the doctor or whatever and it's a bit embarrassing really. (F/FP/late 20 s/-).

They could also stem from respondents' original reasons for going for testing. For example, respondents who wanted to go for testing because they had had unprotected sex, and were ashamed about this, were often concerned that their healthcare provider would judge them and their behaviour as 'shameful'.

Respondents without symptoms often indicated that while testing was a positive practice to engage in, it was not an urgent one.

I went about a year later. There wasn't any cause for concern. There was nothing obvious happening. (M/SH/early 20 s/+).

Lack of urgency here was connected to feelings of invulnerability, feelings that the individual could not really be at risk if everything appeared to be functioning normally.

I would have had unprotected sex with men before but I just assumed I was fine. I presumed it was perfect, I was grand. This kind of immortality vibe, you're grand, you're healthy, everything is fine. (F/FP/late 20 s/+).

The cost of attending a health service for STI testing could also discourage respondents from seeking immediate help.

I mean you could go to your GP and maybe get something done quicker but I went to the clinic where it was free because I couldn't afford to pay whatever to your GP like 50 or 60 euro to see him on top of whatever it costs to get those tests done. (F/STI/late 20 s-).

Several respondents from poorer areas noted that they needed to spend several weeks saving up enough money to pay for their STI tests. In Ireland individuals whose income falls below a certain level can apply for 'medical cards', which entitle them to free healthcare at their local GP practices. Respondents from poorer backgrounds who had these cards, however, often did not want to seek treatment from their local GP because of concerns that they would be judged by him or her as being promiscuous or immoral. As such they sometimes sought treatment at practices or centers where they had to pay for treatment but where they would be unlikely to encounter a healthcare professional who could judge them.

Respondent: I needed to save up the money. It was like 150 euro. I didn't have the money straight away. I got it about a month after that.

Interviewer: Was it difficult to get the money together?

R: Yeah. Cos it was 150 euro because I got a full test.

I: So how did you get the money together.

R: Working. I work like.

I: Did you have to give up stuff.

R: Yeah, it was hard. It was just hard to keep all my money and not spend money. (F/FP/late teens-).

Preferences for attending clinics that were further away from where respondents lived or worked and which were subsequently more difficult to attend were more likely to lead to procrastination behaviors. Clinic opening hours – especially for STI clinics – could also lead to delayed attendance. Respondents who attended these clinics did so for two principle reasons: the clinics were free and they were thought to have expertise in treating STIs. The demand placed on these clinics was such, however, that respondents often faced difficulties accessing them, which further delayed testing.

I was told to be at the clinic 90 minutes before they started testing at 1 pm but I was there an hour early. They'd given out all the tickets at 11.30 am so I was too late. I then asked about the the other hospital clinic and was told about the six week wait. (Male/MSM/late 20 s/-).

Interestingly, some respondents who delayed testing (for reasons unrelated to difficulties accessing clinics because of long-waiting times) did not view themselves as delaying. These respondents appeared to have an elastic conception of what immediacy was in relation to STI-related health-seeking.

Respondent: I went straight away [for test].

Interviewer: And how long was that?

I: I think within four weeks (F/SH/early 20 s/-).

### Re-testing

A minority of respondents (all either women or MSM) attended health services for re-testing. There were four reasons why respondents went for re-tsting. One was to receive a test of cure in the event that they had tested positive for an STI.

They do advise after you've had a course of meds and taken them properly, to go back after six months and have another check. It's all free of charge. (F/GP/late 20 s/+).

The second was to manage any uncertainty remaining from the first test. Uncertainty related to the STI status of the body (whether the individual was positive or negative) and could develop because the individual had not successfully completed his or her course of medication or because her health service had not contacted her to tell her that her results were either positive or negative.

I was wanting to [get re-tested]. I think because I'd been nearly caught, until I'd got the all clear again I wasn't happy. It was more a thing that I needed to do for myself. I was happier once we'd had the all clear. I just needed to get it right in my own mind. (F/GP/late/+).

I needed to see it in black and white 12 months later that it was clear on both of our parts. I think it's just a psychological thing. (F/GP/late 20 s/+).

The third reason related to the cleansing nature of STI testing. Some respondents experienced STI testing as a moral practice (using words like it was the 'right' thing to do, a 'good' thing to do) and received positive feedback from their (negative) test results, which encouraged them to seek further testing in future.

I'm just addicted I think to getting checked. Even though I know I don't have anything it's just nice to know I'm after getting it done again and I'm still clean. (M/MSM/late 20 s/-).

The fourth reason was relevant to re-testers who had tested positive in their first test. Testing positive damaged these respondents' protective cocoons, the psychological filters that they used to screen out risk anxieties; as such they perceived themselves as being more vulnerable to risk than other respondents. STI re-testing, as well as being a course of action that was sometimes advised by the health provider, provided respondents' with a mechanism to manage risk, acting as a psychological 'protective shield' against feelings of vulnerability. Re-testing also had the advantage of enabling these respondents to test their partners' faithfulness, and whether they could continue to trust them or not.

Generally, re-testers found re-testing to be a more positive experience than the initial testing experience. Re-testers had experience of the most effective procedures to use to access particular healthcare settings (for example, knowing to queue early to get into an STI clinic) and their concerns about stigma and being judged had often been positively addressed by healthcare professionals on the first visit.

I just kind of knew what to expect. I had the feelings before so they weren't fresh. They didn't stick in my mind. It's not that it was fine, it was still. It was just a more comfortable experience because I knew what to expect. I knew what was happening. (F/GP/mid 20 s/-).

I would say that it's much easier since I've got it done. It's still, taking off your clothes and stuff like that, a very intimate procedure but the first one is definitely the hardest. (F/FP/late 20/+).

One re-tester's identity concerns became more acute the more she attended health services for testing, however. She felt that regular re-testing signaled to healthcare workers that she was being continuously promiscuous, sleeping with many different risky men on an on-going basis. As such the act of re-testing risked stigmatizing and spoiling her identity more than the original act of testing did.

You know if you change partners then it's like I'm being really bold. It's the whole slut factor again you know. Six months later you have another new partner and you go in for another screening and then it's again the whole what are they going to think of me. (F/GP/late 20 s/-).

## Discussion

As far as we are aware this is the first study to explore the reasons why young adults in the Republic of Ireland go for STI testing, and one of the few studies to explicitly focus on and explore this topic in the international literature [8]. Previous studies found that individuals attend health services for STI testing for a variety of reasons including: the desire to be free of contamination [8], health maintenance, the presence of symptoms (own and partner's) and partner's risk status [7]; and the individual's own perceived level of risk [16]. Some of the reasons for seeking a STI test that were revealed in our study have been identified in previous studies, notably having had unprotected sex and anxieties around symptoms [7,8]. We believe that this is the first study to identify the importance of transitional moments in young adults' lives as prompts for their seeking STI testing. We also believe that we are the first to identify the ritualistic identity benefits that testing at transitional moments can supply to young people, though similar benefits have been observed in relation to the re-testing process in a study by Hilary Piercy [14]. Piercy's study found that re-testing is often used as a practice to signal that the individual is at the end of a dirty stage in his or her life and about to begin a new, uninfected clean stage.

A number of the factors encouraged respondents in this study to seek STI testing (including partner's perceived risk status, viewing testing as a positive practice). Several of these have previously been reported [17,18]. The specific concerns expressed about Chlamydia (as a prompt to full STI screening), articulated by most of our respondents, may be particular to this study. These concerns may reflect growing awareness and concern amongst young adults in Ireland about Chlamydia, as an outcome of health promotion and awareness activities by the health services, voluntary agencies and the media in Ireland. The increased prevalence and reported incidence of Chlamydia may also mean that young people are encountering more other young people with the infection, an interaction that could disrupt the feelings of invulnerability that young people might otherwise experience in relation to STIs.

Respondents indicated that a number of barriers (stigma, embarrassment, shame and cost) often deterred them from immediately following through on their decision to go for STI testing. These findings echo previous research [19-21]. Such delays are worrying because young people who are unknowingly positive for STIs may transmit the infection to others while they consider when and where they will attend health services for testing. Infection duration is closely correlated with the likelihood of harmful consequences to self and others [22]. One of the more interesting findings from this study, however, is the realization that some young people who delayed seeking testing for several weeks or even months not see this as delay. It might be useful for health promotion efforts (which appeared to have a significant effect on at least some of our respondents) to therefore include messages highlighting that STIs can have deleterious consequences on individuals soon after transmission, and to communicate in straight forward terms that social and biological concepts of immediacy can be quite different.

Limitations of the study include the size of the study sample (drawn from several clinical settings, and from two regions of Ireland), and the limited number of men who took part in the study. Because of the small sample size the results must be considered to be tentative. A second limitation is that 80% of the interviews were conducted by telephone with only 20% conducted face-to-face. Telephone interviews might be less well-controlled (e.g. participants may not have been as free to talk as they might have been in a clinical or research setting), and the interviewer did not have access to non-verbal responses to guide follow-up questioning. It is therefore possible that additional information might have emerged if the interviews were conducted face-to-face rather than over the telephone.

However, our recruitment of young adults from a number of settings (see table 1) and from two regions of the country is likely to have increased the representativeness of the findings as those of young adults in Ireland. Because of time constraints in the interviews and the willingness of young people to explore the issues that were the main objective of the study, we did not investigate STI-related health seeking behaviors not associated with attending a health service for testing, such as seeking health advice on the internet. This is an important area for future research to address as young adults may engage in a variety of help-seeking activities either as a preliminary to, or in parallel with, health service attendance [22].

## Conclusion

The findings of this study suggest that there are multiple pathways, catalytic and inhibitory factors that can lead, delay and possibly deter young adults from seeking STI testing. These should be viewed, explored and exploited as opportunities for public health and health service interventions. Health promotion efforts to encourage STI testing should promote the STI testing-enablers identified here, reinforcing young people's views that the risk of acquiring a STI through unprotected sex is real; that STI testing is a 'mature' responsible practice and that it would be helping not just young people but also their friends and partners; and reassuring them (assuring that) young people will be judged positively by healthcare providers when they go for testing. Given respondents' stigma-related concerns health promotion materials should also stress the confidentiality of STI testing services. It follows from our findings that health promotion messages should be directed at four specific groups: those leaving and/or entering relationships where condoms have not or will not be used, those who have had unprotected sex and those with STI-related symptoms. Health promotion messages should also emphasise that STIs begin to have an impact upon individuals and can be transmitted to others from shortly after their acquisition. This might help to combat the worrying delays in testing reported in this paper. While there might be some risk of encouraging over-testing in a small number of people, young people's notion of STI testing as a cleansing action should be seen as an opportunity to frame positive messages that build upon those key transition windows of opportunity revealed in these enlightening interviews with young people.

## Competing interests

The authors declare that they have no competing interests.

## Authors' contributions

MB and RB both analyzed the interview transcripts. MB wrote the first draft and RB revised it. Both authors read and approved the final manuscript.

## Pre-publication history

The pre-publication history for this paper can be accessed here:

http://www.biomedcentral.com/1471-2458/9/311/prepub
